# COVID-19 and Cold Agglutinin Hemolytic Anemia

**DOI:** 10.1055/s-0040-1715791

**Published:** 2020-08-20

**Authors:** Diana V. Maslov, Victoria Simenson, Suma Jain, Ambuga Badari

**Affiliations:** 1 Department of Internal Medicine, Ochsner Clinic Foundation, New Orleans, Louisiana, United States; 2 Department of Pulmonary and Critical Care, Ochsner Clinic Foundation, New Orleans, Louisiana, United States; 3 Department of Haematology and Oncology, Ochsner Clinic Foundation, New Orleans, Louisiana, United States

**Keywords:** viral infection, thrombosis, autoantibodies

## Abstract

Novel coronavirus disease 2019 (COVID-19) has spread throughout the world and has infected close to 4 million people. It commonly presents with fever, cough, and fatigue. Due to the high inflammatory response, it is suggested that the coagulation cascade is enhanced causing thrombotic events for many patients. We describe a patient with clinical features of cerebrovascular accident, as well as documented blood clots in bilateral upper extremities. Labs revealed the presence of cold agglutinin hemolytic anemia. The association between cold agglutinin autoimmune hemolytic anemia and thrombotic events in COVID-19 patients has not been well investigated. The patient unfortunately passed away within 48 hours after admission. This case stresses the importance of considering a full workup to diagnose autoimmune hemolytic anemia (AIHA) in COVID-19 patients with thromboses and possible implications for management.

## Introduction

Starting in November, 2019, novel coronavirus disease 2019 (COVID-19) emerged in Wuhan, China, and by March of 2020, spread worldwide. Currently, there are over 3,800,000 cases with 268,000 deaths. 1 Patients present with fever, cough, myalgias, and fatigue. 2 Anosmia and ageusia have also been found to be common symptoms. 3 As COVID-19 emerged, many patients also presented with coagulation disorders. 4 It is presumed that the high levels of inflammation throughout the body cause excessive activation of the coagulation cascade. Hospitals have been using anticoagulation aggressively to mitigate the catastrophic thrombotic events. Even with this approach, a high number of patients with COVID-19 develop life-threatening thrombotic complications. 5

Although many COVID-19 patients present with coagulopathy, the relationship between COVID-19 and autoimmune hemolytic anemia (AIHA) causing thrombosis has not been well investigated. Hemolytic anemia presents with anemia, elevated lactate dehydrogenase (LD) and bilirubin, and decreased haptoglobin. 6

This case discusses a patient with no history of autoimmune disease presenting with COVID-19 who quickly deteriorated and ultimately passed away due to thrombotic complications. Tests later revealed cold agglutinin hemolytic anemia.

## Case Report

A 48-year-old African American male with essential hypertension, insulin-dependent diabetes mellitus, obesity (body mass index [BMI] = 36.2 kg/m ^2^ ), and end-stage renal disease on peritoneal dialysis presented to the emergency department with a weeklong history of weakness, anosmia, and decreased appetite. Review of systems and physical exam were limited due to isolation precautions but were positive only for chronic bilateral leg numbness and weakness. Vitals were remarkable for elevated blood pressure requiring intravenous nicardipine. COVID-19 test was positive. The patient's oxygen saturation was 100% on room air while being admitted. During his admission, it was noted that all blood draws (from numeral sites, including arterial sticks) were clotting in the syringes as it was being drawn for laboratory investigations. Computed tomography (CT) of the head without contrast, as well as chest X-ray, showed no acute findings, and the patient was admitted to the medical intensive care unit (MICU) on intravenous nicardipine and heparin.

After 3 hours of admission, his neurological status declined and he was intubated for airway protection. He became hypotensive requiring vasopressors. Venous Doppler ultrasound of upper extremities revealed clots in bilateral upper extremities. Due to difficulty obtaining laboratory results, it took a few hours to discover that the patient had a hemoglobin of 4.5 g/dL (normal range: 14.0–18.0 g/dL), platelets of 135,000 (normal range: 150,000–400,000), haptoglobin of 21 mg/dL (normal range: 30–250 mg/dL), LD of 2,832 U/L (normal range: 110–260 U/L), and total bilirubin of 2.5 mg/dL (normal range: 0.1–1.0). Fibrinogen was 478 mg/dL (normal range: 182–366 mg/dL), making disseminated intravascular coagulation (DIC) unlikely. D-Dimer was 15.67 mg/L (normal: < 0.5). Anticardiolipin antibody (ACA) immunoglobulin (Ig)-M was slightly elevated at 14.2 (normal range: 0–12.49). ACA IgG, Beta-2 glycoprotein IgG, IgM were negative.

Tests for cold agglutinin were sent and the patient received blood transfusions in the setting of hemolytic anemia.

Overnight, the patient continued to have worsening neurological status in which his pupils became fixed and dilated on physical exam. Although he was too unstable for repeat head imaging, it was determined that the patient likely had an intracerebral insult. After discussions with the family, comfort care was initiated and the patient expired shortly thereafter. Laboratory results later confirmed cold agglutinin antibody (titer > 1:512) and direct coombs positive.

## Discussion

There is a high incidence of thrombotic events in COVID-19 patients. 4 DIC appears to be common in critically ill COVID-19 patients. 7 However, other contributing factors for thrombosis, including cold agglutinin disease, have to be considered. Multiple studies have seen an association between cold agglutinin disease and thrombotic events. 8 Chapin et al discussed the increased risk of thrombotic events in those with cold agglutinin disease. 9 Bleakly et al showed a relationship between DIC and cold agglutinin hemolytic anemia. 10

Cold agglutinins are formed when B cells produce IgM autoantibodies with an antigen antibody reaction between 0 to 4°C. These autoantibodies are capable of agglutinating red blood cells, causing complement-mediated extravascular hemolysis and resulting in an AIHA. When cold agglutinin autoantibodies are produced in association with an underlying hematologic malignancy or infection, this is called cold agglutinin syndrome. 11

Patients with cold agglutinin syndrome present with laboratory evidence of hemolysis (high LD, high bilirubin, and low haptoglobin) and positive direct coombs test. 6 Clinically, they can present with acral ischemia, especially after exposure to cold. Secondary cold agglutinin syndrome is often associated with infections, including *Mycoplasma pneumoniae* , Epstein–Barr virus, and cytomegalovirus. 12 There is evidence that cold agglutinin syndrome has also been seen in COVID-19 patients. 13 Diagnosis of cold agglutinin mediated hemolytic anemia is made when a patient has evidence of AIHA, positive direct coombs test, and positive cold agglutinin titer.

In this case, the patient did not have a complete evaluation for underlying hematologic malignancy. Antiphospholipid antibodies were negative. While it is likely that the cause of this patient's cold agglutinins was COVID-19 infection, this cannot be definitive without a full workup. Another publication describes similar case presentations in which an underlying B-cell lymphoma was identified. 14

No systematic studies have been performed to determine optimal treatment for cold agglutinin syndrome, due to its rarity. The primary goal is to treat the underlying infection or malignancy. Case reports have reported using corticosteroids to treat *M. pneumoniae* associated cold agglutinin syndrome. 15 However, less is known on how to treat patients with severe, life-threatening hemolytic anemia with cold agglutinins. Plasmapheresis and intravenous immunoglobulin have been used in the setting of fulminant hemolytic anemia due to cold agglutinin disease 15 and could have been considered in this case. Unfortunately, the cold agglutinin test did not result until after the patient's death. It may have been beneficial to empirically treat this critically ill patient with multiple signs of cold agglutinin AIHA. In particular, the patient was noted to have clotted blood in all initial laboratory draws.

## Conclusion

When a patient presents with thrombosis, it's important to consider an underlying hemolytic disease. One may want to complete a full work up for AIHA and consider beginning treatment depending on the acuity of the condition.
